# Redefining Difficult-to-Treat Systemic Lupus Erythematosus: Biomarkers of Molecular Refractoriness Beyond Clinical Failure

**DOI:** 10.3390/ijms27094026

**Published:** 2026-04-30

**Authors:** Agata Matusiewicz, Alicja Paś, Sylwia Wiktorzak, Marzena Olesińska

**Affiliations:** Department of Connective Tissue Diseases, National Institute of Geriatrics, Rheumatology and Rehabilitation, 02-637 Warsaw, Poland; agata.matusiewicz@spartanska.pl (A.M.); sylwia.wiktorzak@spartanska.pl (S.W.);

**Keywords:** systemic lupus erythematosus, biomarkers, interferon pathway, gene expression profiling, precision medicine, treatment resistance, lupus nephritis, CAR-T

## Abstract

Difficult-to-treat systemic lupus erythematosus (D2T-SLE) remains a major unmet challenge in contemporary lupus care, yet it continues to be defined predominantly by clinical non-response rather than underlying biology. Current biomarkers largely quantify inflammatory burden, immune complex activity, or organ damage and do not reliably capture persistent activation of pathogenic pathways under therapy. Emerging multi-omics, single-cell, and longitudinal studies suggest that, in a subset of patients, apparent treatment failure may reflect incomplete attenuation of dominant immune circuits rather than uniformly elevated inflammation. We propose molecular refractoriness in systemic lupus erythematosus (SLE) as sustained, pathway-level immune activity despite apparently adequate, mechanism-directed therapy. We outline the major immune programs implicated in this process—including interferon-enriched, B-cell/plasmablast-associated, neutrophil extracellular trap (NET)-related, cytotoxic T-cell, and cytokine-associated states—and discuss their relevance for biomarker development and precision trial design. Importantly, we emphasize that interferon gene signatures (IGS) should be interpreted as context-dependent and non-specific markers of interferon responsiveness, reflecting combined activity of type I, II, and III interferons, and functioning primarily as predictive rather than mechanistic biomarkers. We further highlight critical limitations of a purely endotype-based model, including the need to distinguish true molecular refractoriness from damage-dominant and pseudo-refractory states, as well as the emerging role of immune-reset strategies such as cluster of differentiation 19 (CD19)-directed chimeric antigen receptor T-cell (CAR-T) therapy, which may overcome refractoriness independently of specific pathway dominance. These observations suggest that difficult-to-treat SLE encompasses biologically heterogeneous states that may not be fully captured by pathway-resolved stratification alone. Reframing D2T-SLE as a biologically heterogeneous state of incomplete immune attenuation may help bridge the gap between clinical treatment failure and mechanism-informed precision medicine in systemic lupus erythematosus.

## 1. Introduction

Systemic lupus erythematosus (SLE) is a clinically and immunologically heterogeneous autoimmune disease characterized by marked variability in immune pathway activation, organ involvement, and treatment response. Over the past decade, advances in immunopathogenesis-informed therapies, together with the implementation of treat-to-target (T2T) strategies, have led to measurable improvements in disease control and long-term outcomes [1,2,3]. Within this framework, remission and low disease activity have emerged as key therapeutic goals [4]. Remission, as defined by the Definitions of Remission in Systemic Lupus Erythematosus (DORIS) task force, requires the absence of clinical disease activity (clinical Systemic Lupus Erythematosus Disease Activity Index (SLEDAI) = 0), a Physician Global Assessment (PGA) < 0.5, and prednisolone ≤ 5 mg/day, with stable background immunosuppressive therapy permitted [5]. In contrast, the Lupus Low Disease Activity State (LLDAS) represents a more attainable target, defined by SLEDAI ≤ 4 without major organ activity, no new disease activity, PGA ≤ 1.0, and prednisolone ≤ 7.5 mg/day [6]. Both states have been consistently associated with reduced flare rates and decreased damage accrual over time, and are linked to improved treatment outcomes, including lower glucocorticosteroid (GCS) exposure [2,6,7,8]. Current treatment paradigms, as reflected in international recommendations including the 2025 American College of Rheumatology (ACR) and European Alliance of Associations for Rheumatology (EULAR) guidelines, remain primarily anchored in clinical phenotype and organ involvement, emphasizing universal use of hydroxychloroquine (HCQ) and minimization of GCS exposure [9,10]. However, these approaches do not yet incorporate molecular stratification or formally integrate DORIS or LLDAS definitions as operational decision-making tools [11].

Despite these advances, a substantial proportion of patients fail to achieve or sustain remission or low disease activity. Persistent symptoms, recurrent flares, and ongoing GCS dependence remain common, even in the era of biologic and targeted therapies [12,13,14]. Real-world data indicate that remission is less frequently attained than LLDAS, with patients exhibiting persistent mucocutaneous and musculoskeletal manifestations disproportionately represented among those with suboptimal disease control [8,12,13].

These observations have led to the emergence of the concept of difficult-to-treat SLE (D2T-SLE) [15]. However, failure to achieve therapeutic targets does not in itself explain the biological basis of treatment resistance. Current definitions of refractoriness, largely grounded in clinical activity and treatment exposure, do not distinguish between persistent targetable inflammation and alternative drivers of disease burden, including irreversible organ damage, comorbid conditions, treatment non-adherence, or incomplete biological response under therapy [15].

This distinction is central to the present review. We propose that, in a subset of patients, difficult-to-treat disease reflects sustained activity of dominant pathogenic immune programs despite apparently adequate, mechanism-aligned therapy. In this context, molecular refractoriness may provide a more biologically coherent framework for interpreting treatment failure by linking persistent clinical disease to incomplete suppression of specific pathogenic pathways under therapeutic pressure.

## 2. From Clinical Non-Response to Molecular Persistence: What Does “Difficult-to-Treat” Truly Represent?

The term D2T-SLE is commonly applied to patients with persistent clinical manifestations despite multiple therapeutic interventions [15]. However, clinical refractoriness should not be assumed to reflect ongoing targetable immune activation. Composite disease activity indices may not reliably distinguish reversible inflammatory activity from irreversible organ damage [16]. Instruments such as SLEDAI integrate clinical and serological features into global activity scores, but remain limited in sensitivity and in their ability to resolve active immunopathology from cumulative tissue injury across organ systems [16].

This distinction becomes particularly important in lupus nephritis, where persistent proteinuria may represent either ongoing immune-mediated renal inflammation or chronic structural remodeling, and where histopathologic activity may diverge from conventional clinical readouts [17]. Because damage accrual is independently associated with both cumulative inflammatory burden and GCS exposure [18,19], misclassification of chronic injury as active disease may lead to inappropriate therapeutic escalation and further non-inflammatory harm.

GCS use remains common in patients perceived as difficult to treat [19,20]. Yet prolonged glucocorticosteroid exposure should not be interpreted as a surrogate for persistent immune pathway activation. Cohort data demonstrate a dose-dependent association between GCS exposure and irreversible organ damage [19], independent of clinical and serological activity [20]. Continued GCS reliance may therefore amplify damage accrual even in the absence of sustained inflammatory progression, further complicating the interpretation of apparent treatment failure.

Refractory disease in SLE is unlikely to reflect a single biological process [15,21]. Rather, it may encompass mechanistically distinct non-response states driven by differential dominance of inflammatory circuits, including type I interferon signaling [22,23], B-cell activating factor (BAFF)-dependent B-cell activation [24,25], T helper 17 (Th17)-associated immune programs [26], neutrophil extracellular trap (NET)-linked vascular inflammation [27]. Circulating cytokines and B-cell-related biomarkers may support partial stratification of disease activity and therapy-associated response patterns [28,29,30]. However, molecular profiling is not incorporated into current D2T definitions, and no validated framework currently distinguishes persistent pathway activation from clinical non-response alone.

## 3. The Biomarker Paradox in SLE: Measuring Inflammation Without Capturing Refractoriness

Over the past decade, biomarker discovery in SLE has generated an increasingly complex repertoire of serological, cytokine-based, transcriptomic, and organ-resolved markers [29,30,31,32]. Collectively, these biomarkers have improved the quantification of inflammatory burden, facilitated the evaluation of organ involvement, and, in selected contexts, informed therapeutic response stratification [28,29,30,31]. However, despite their growing analytical sophistication, most currently available biomarkers remain surrogates of inflammatory activity, immune complex turnover, or tissue injury rather than direct indicators of persistent, treatment-resistant immune pathway activation [29,30].

Accordingly, the current biomarker landscape in SLE largely addresses two clinically relevant questions: whether inflammatory disease activity is present and which organ system is involved. It does not reliably address a third question that is central to difficult-to-treat SLE: whether the dominant pathogenic circuit remains biologically active despite apparently adequate, mechanism-directed therapy [15,29,30]. This distinction is not semantic; it is biologically consequential. Biomarkers that capture inflammatory burden or organ-specific injury [29,30] and, in selected contexts, predict therapeutic response [28] do not, by themselves, define molecular refractoriness.

Table 1 summarizes selected biomarkers according to their principal clinical or biological readout and highlights the limited evidence supporting their utility in the assessment of treatment refractoriness. Collectively, these biomarkers illustrate a central paradox in SLE: although the field is increasingly enriched with markers of inflammatory activity, tissue injury, and organ involvement, it remains markedly deficient in validated tools capable of identifying persistent immune circuit activation under therapy. An expanded overview of currently available biomarkers and their limitations in the context of molecular refractoriness is provided in Supplementary Table S1.

This overview underscores a fundamental imbalance in the current biomarker repertoire of systemic lupus erythematosus. Most available biomarkers quantify inflammatory burden or reflect organ-specific immune involvement [29,30,31], whereas only a limited subset informs therapeutic response stratification [28]. Notably, even these response-associated markers do not capture the biological mechanisms underlying treatment failure.

A central limitation of the current biomarker landscape is its inability to determine whether dominant pathogenic pathways remain active despite apparently adequate, mechanism-directed therapy [29,30]. Consequently, existing biomarkers primarily reflect disease activity, tissue injury, or likelihood of response rather than persistent immune pathway activation under treatment [29,30,31]. This limitation has important clinical implications, as it constrains the ability to distinguish ongoing, targetable inflammation from non-inflammatory drivers of disease burden, including irreversible damage or comorbidity.

In this context, difficult-to-treat SLE remains largely defined by clinical features and treatment history rather than underlying biological mechanisms [15]. The absence of validated biomarkers capable of capturing pathway persistence under therapy represents a major unmet need. Future progress will depend on moving beyond static measures of disease activity toward longitudinal, pathway-resolved approaches capable of identifying immune programs that remain insufficiently suppressed under therapy.

## 4. Beyond Activity and Damage: Toward Pathway-Resolved Biomarkers of Molecular Refractoriness

Traditional SLE biomarkers remain clinically useful, but they primarily capture inflammatory burden or cumulative organ damage rather than biological treatment resistance. However, they do not explain why a subset of patients progresses toward difficult-to-treat disease despite apparently adequate, mechanism-aligned therapy. Emerging multi-omics and single-cell datasets suggest that clinical non-response may, in some patients, reflect incomplete suppression of dominant immune circuits rather than persistently elevated global inflammation [32,41].

Systemic lupus erythematosus comprises biologically heterogeneous inflammatory endotypes in which distinct immune pathways predominate across individual patients [32,42]. Longitudinal blood-based and tissue-resolved analyses have demonstrated sustained activation of discrete transcriptional modules in subsets of non-responders, indicating persistence of specific immune axes under therapeutic pressure [43,44,45]. These immune programs are not static; their relative dominance may shift over time with disease evolution and treatment exposure [42,45].

The pathways most consistently implicated include interferon-associated immune activation, humoral immune activation characterized by plasmablast expansion and autoreactive antibody-secreting cell signatures [43,46], neutrophil- and NET-associated transcriptional programs linked to innate immune activation and vascular injury [33], and cytotoxic T-cell-associated gene signatures observed in severe systemic phenotypes [32,41]. By contrast, tissue-remodeling and fibrosis-associated signals detected in non-responding organ compartments particularly in lupus nephritis may reflect structural remodeling or accrued damage rather than persistent pharmacologically suppressible immune activity and should therefore be interpreted separately from immunologically defined refractory states [45]. Clinically, this distinction matters because damage-dominant states may call for restraint rather than further immunosuppressive escalation. Notably, the interpretation of interferon-related signals requires particular caution. Although elevated interferon-stimulated gene expression (interferon gene signature IGS) has historically been used as a proxy for interferon pathway activation in SLE, accumulating evidence indicates that IGS should not be interpreted as a specific surrogate of type I interferon (IFN-I) activity alone. Interferon-responsive transcriptional programs may reflect the combined and context-dependent effects of type I, type II, and type III interferons, particularly in severe systemic disease and lupus nephritis [22,47]. Moreover, discordance between elevated IGS and directly measurable circulating IFN-I levels has been observed, suggesting that IGS reflects a broader downstream state of interferon responsiveness rather than isolated IFN-I pathway dominance [47]. Accordingly, interferon-related endotypes may be more appropriately conceptualized as interferon-enriched states rather than as definitively IFN-I-driven processes. This distinction has direct translational implications: if the biomarker used to define an endotype is not mechanistically specific to the therapeutic target, the rationale for strict pathway-to-therapy matching becomes less secure. IGS is therefore better understood as a predictive or enrichment biomarker than as mechanistic evidence of pathway dominance. Within this framework, biomarkers must evolve from static indicators of activity or damage toward longitudinal, pathway-resolved tools capable of detecting incomplete attenuation of dominant immune modules. Importantly, single downstream biomarkers—particularly transcriptomic signatures—may be insufficient to define mechanistic pathway dominance in isolation. More informative biomarker models will likely require integration of transcriptomics with direct cytokine measurements, cell-resolved immune profiling, and, where relevant, tissue-level data.

The resulting translational framework is outlined in Table 2.

The table summarizes proposed immune endotype patterns associated with persistent pathway activity, their dominant biological circuits, representative molecular signals, potential clinical correlates, and possible precision-therapy implications in difficult-to-treat SLE.

Recent transcriptomic studies further support the concept that therapeutic non-response in SLE may reflect persistence of dominant immune circuits rather than nonspecific inflammatory activity. In patients who failed to achieve LLDAS following cyclophosphamide, rituximab, or belimumab, three reproducible molecular endotypes were identified: a PD-1/DNA repair-enriched T-cell-dominant profile, a cytokine-driven IL-6/IL-17-high/IL-2-low signature, and an inflammasome-associated phenotype, with robust discriminatory performance (AUC 0.889) [48]. Transcriptome-reversal modeling further suggested therapeutic alignment, predicting cluster of differentiation 19 (CD19)-directed chimeric antigen receptor T-cell (CAR-T) therapy (CD19 CAR-T) sensitivity in the T-cell-dominant endotype and potential benefit from IL-17/IL-6 blockade or low-dose IL-2 in cytokine-driven disease [48].

Randomized trials further support the principle that clinical efficacy may depend, at least in part, on the underlying immune program. Belimumab improved SRI-4 responses in BLISS-52/76 and enhanced renal outcomes in BLISS-LN [49,50]. Similarly, anifrolumab increased British Isles Lupus Assessment Group-based Composite Lupus Assessment (BICLA) responses and LLDAS attainment, with greater benefit observed in patients with a high interferon gene signature [40]. Importantly, this association is best interpreted as evidence of predictive enrichment for interferon-pathway-directed therapy rather than as mechanistic confirmation of isolated IFN-I pathway dominance, given the limited biological specificity of IGS [40,47].

**Figure 1:**
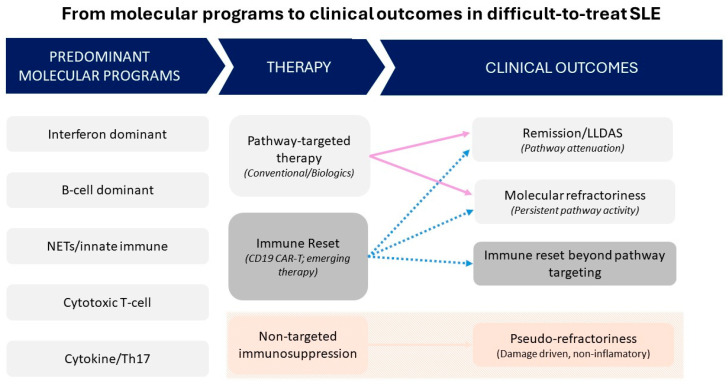
Conceptual framework linking predominant molecular programs, therapeutic strategies, and clinical outcomes in difficult-to-treat systemic lupus erythematosus (D2T-SLE). Distinct molecular programs underlie heterogeneous treatment responses in D2T-SLE. Pathway-targeted therapy may result in remission or LLDAS when the dominant immune circuit is adequately suppressed, whereas incomplete attenuation leads to molecular refractoriness. In the absence of a dominant inflammatory program, non-targeted immunosuppression may result in pseudo-refractory, damage-driven states. Immune-reset strategies may overcome refractoriness independently of pathway-specific targeting. Dashed arrows indicate transitions between states.

Collectively, these findings support a conceptual shift from activity-based treatment escalation toward molecularly stratified intervention and position immune endotyping as a potential framework for biologically guided management of difficult-to-treat SLE [51]. The proposed endotype patterns require prospective validation and should be regarded as a hypothesis-generating framework rather than a definitive classification system. The conceptual relationships between predominant molecular programs, therapeutic strategies, and clinical outcomes in difficult-to-treat SLE are summarized in Figure 1.

## 5. Defining Molecular Refractoriness: Toward Operational and Longitudinal Criteria

If refractory SLE reflects persistence of a dominant immune pathway, its definition should extend beyond baseline molecular abnormalities alone. In this context, molecular refractoriness may be conceptualized as a dynamic state of incomplete attenuation of a biologically coherent inflammatory module despite apparently adequate, mechanism-directed therapy [32,41]. This perspective is broadly aligned with biomarker qualification principles outlined in the National Institutes of Health/Food and Drug Administration Biomarkers, EndpointS, and other Tools (NIH/FDA BEST) framework T framework, which emphasize biological plausibility, longitudinal validation, and clinical relevance [52].

At the same time, any framework for molecular refractoriness presupposes exclusion of common non-immunologic causes of apparent treatment failure. In routine clinical practice, persistent symptoms or failure to achieve treatment targets may reflect suboptimal adherence, including subtherapeutic HCQ exposure, infection, irreversible organ damage, or comorbid conditions such as fibromyalgia, rather than ongoing targetable immune activity [9,53]. Failure to distinguish these states risks misclassifying pseudo-refractory disease as biological refractoriness and may drive inappropriate escalation of immunosuppression. Accordingly, exclusion of non-inflammatory and non-adherence-related contributors should be considered a prerequisite for any biology-based interpretation of difficult-to-treat SLE.

Four provisional criteria may help operationalize this concept.

**Mechanistic specificity**

Molecular refractoriness should be anchored to persistence of a reproducible, pathway-resolved module linked to a defined immune axis rather than to isolated elevation of a single biomarker [32,41]. Importantly, downstream biomarkers such as transcriptomic signatures may support pathway attribution but do not necessarily establish mechanistic specificity in isolation.

**Temporal persistence**

Sustained module activity across sequential measurements obtained within an appropriate therapeutic window may indicate incomplete pathway attenuation rather than transient biological fluctuation [47,48]. However, prospective validation and quantitative thresholds remain to be established.

**Adequate pathway-directed exposure**

Interpretation presupposes documented exposure to a therapy capable of modulating the implicated pathway. Persistence of pathway-associated signals under such conditions may suggest incomplete biological suppression. Observed associations between interferon-pathway activity and response to interferon blockade, as well as between B-cell-related biomarkers and B-cell-directed therapies, support the plausibility of mechanism-specific assessment, although currently available data remain exploratory [25,28,40].

**Clinical correlation**

Persistent molecular activity should be interpreted alongside validated clinical outcomes such as relapse, GCS dependence, or damage accrual [2,5,6]. In the absence of clinical instability, molecular persistence alone warrants cautious interpretation.

Within this framework, a pre-refractory state may be conceptualized as persistent or recurrent pathway-level molecular activity preceding overt clinical treatment failure. Although this concept remains hypothetical, emerging longitudinal transcriptomic and single-cell studies suggest that sustained immune signaling—particularly within interferon-associated pathways—may persist in some patients before clinically apparent non-response becomes fully established [44,45]. Clinical remission should not be assumed to indicate durable biological control, as persistent molecular activity may precede relapse and reflect early pathway-level refractoriness, although this hypothesis requires prospective confirmation [12]. In TULIP-2, the greater benefit of anifrolumab among patients with a high interferon gene signature illustrates that baseline pathway activity may inform therapeutic responsiveness [40]. However, as discussed above, such biomarkers should be interpreted primarily as predictive or enrichment markers rather than as mechanistically definitive indicators of isolated pathway dominance [47].

Taken together, accumulating translational evidence suggests that molecular refractoriness may be better understood as insufficient attenuation of dominant inflammatory programs rather than as a set of discrete static phenotypes [32,41,42,48]. At present, however, this framework remains conceptual and should be regarded as hypothesis-generating pending formal validation.

## 6. Reframing Difficult-to-Treat SLE: Clinical and Translational Implications

This framework carries several practical implications for both clinical care and trial design. Importantly, current treatment paradigms in systemic lupus erythematosus remain grounded in international guideline recommendations. The EULAR recommendations emphasize universal use of HCQ as background therapy, minimization of GCS exposure, and early introduction of immunomodulatory and biologic agents according to organ involvement and disease severity [10]. HCQ plays a central role not only in reducing flare risk and improving survival, but also in modulating immune activation, including interferon-related pathways, and may influence biomarker profiles relevant to disease monitoring and treatment response [9,10,54]. Escalating GCS or empiric immunosuppression without evidence of pathway attenuation may increase toxicity without meaningfully altering the dominant underlying immune program [20]. Second, longitudinal molecular monitoring may help identify patients with persistent pathway activity before overt clinical deterioration becomes apparent [44,45]. Third, future clinical trials may benefit from stratification according to dominant, non-attenuated inflammatory modules rather than relying exclusively on composite disease activity indices [32,45].

At the same time, not all successful therapeutic strategies in refractory SLE necessarily operate through selective pathway suppression. The emerging efficacy of CD19-directed CAR-T therapy suggests that, in some patients, broad immune reprogramming may overcome refractoriness independently of specific pathway dominance [55,56,57]. Pathway-resolved biomarkers may therefore be most useful where immune activity remains persistent but targetable, whereas immune-reset strategies may become more relevant in deeply entrenched autoreactive states.

Taken together, this model offers a biologically informed complement to existing clinical definitions of difficult-to-treat SLE, but its clinical utility will depend on prospective validation.

## 7. Discussion

Difficult-to-treat systemic lupus erythematosus should not be viewed as a uniform state of uncontrolled inflammation, but as a heterogeneous clinical category arising from distinct biological and non-biological mechanisms. In a subset of patients, persistent disease may reflect incomplete attenuation of dominant immune programs despite apparently adequate, mechanism-directed therapy. In others, apparent refractoriness may instead reflect pseudo-refractory states, accrued organ damage, or tissue remodeling rather than ongoing targetable inflammation.

This distinction has direct implications for both therapeutic strategy and biomarker interpretation. Molecular stratification may help identify patients most likely to benefit from biologically aligned intervention, but it should not be mistaken for a complete model of refractory disease. The emerging success of immune-reset approaches such as CAR-T further suggests that, in some cases, refractoriness may be overcome through broader immune reprogramming rather than selective pathway suppression alone.

Importantly, current treatment recommendations, including the 2025 American College of Rheumatology guidance for SLE management, remain anchored in clinical phenotype, organ involvement, and conventional treatment response rather than molecular stratification. Thus, the concept of molecular refractoriness should currently be regarded as a biologically plausible but still unvalidated translational framework rather than a clinically established classification.

Future work should focus on validating reproducible biomarkers of pathway persistence, defining how such markers interact with pseudo-refractory and damage-dominant states, and determining whether their implementation can improve treatment selection, monitoring, and long-term outcomes in D2T-SLE.

## 8. Materials and Methods

This article is a narrative review and conceptual analysis of the literature addressing biomarkers, immune pathway heterogeneity, and treatment refractoriness in systemic lupus erythematosus, with particular emphasis on difficult-to-treat disease and the emerging concept of molecular refractoriness. The manuscript was based on critical analysis and synthesis of published peer-reviewed literature, including clinical studies, translational investigations, biomarker-focused reports, and selected multi-omics and single-cell studies relevant to persistent immune pathway activation under therapy. Particular emphasis was placed on studies of direct relevance to biomarker interpretation, immune endotyping, and mechanism-informed therapeutic response in SLE.

This manuscript does not report original experimental research involving humans or animals; therefore, ethical approval was not required. No new datasets were generated or analyzed for the purpose of this review, and no accession numbers apply. Generative artificial intelligence was not used to produce scientific content, interpret data, generate figures, or formulate conclusions.

## 9. Conclusions

Difficult-to-treat systemic lupus erythematosus remains defined predominantly by clinical criteria, with only limited integration of molecular stratification into routine decision-making. Emerging transcriptomic and single-cell data suggest that, in a subset of patients, therapeutic non-response may reflect incomplete suppression of dominant immune pathways rather than uniformly elevated inflammatory activity.

Although prospective validation is required, longitudinal, pathway-resolved molecular assessment may help distinguish delayed clinical improvement from sustained biological activity and identify patients at risk of progression toward a pre-refractory state. Such an approach could complement existing treat-to-target strategies by enabling biologically informed reassessment of the prevailing inflammatory axis.

Rather than representing a single state of treatment failure, refractory SLE may ultimately prove to be a biologically heterogeneous condition requiring both molecular resolution and clinical restraint.

For clarity, the key concepts proposed in this review are summarized below:

- Difficult-to-treat SLE represents a biologically heterogeneous condition rather than a uniform state of uncontrolled inflammation.
- Molecular refractoriness may reflect persistent activation of dominant immune pathways despite apparently adequate, mechanism-directed therapy.
- Current biomarkers remain insufficient to capture pathway persistence under treatment, highlighting a major unmet need in SLE.
- Interferon gene signatures should be interpreted as non-specific, predictive biomarkers rather than mechanistic indicators of isolated IFN-I pathway dominance.
- Accurate interpretation of refractoriness requires exclusion of non-immunological drivers, including non-adherence, comorbidities, and irreversible organ damage.
- Emerging immune-reset strategies, such as CD19-directed CAR-T cell therapy, challenge purely pathway-based models and suggest alternative routes to disease control.

## Figures and Tables

**Table 1:** Functional Classification of Biomarkers in SLE and Their Limitations in Capturing Molecular Refractoriness. The table summarizes selected biomarkers according to their principal clinical or biological readout, current evidence for refractoriness assessment, and major limitations in the context of difficult-to-treat SLE.

Domain	Biomarker	Principal Clinical/Biological Readout	Evidence for Refractoriness Assessment	Limitation in the D2T-SLE Context	References
Conventional systemic activity biomarkers	Anti-dsDNA	Correlates with global disease activity, particularly in lupus nephritis	Not assessed for molecular refractoriness	Reflects immune complex-associated activity rather than persistent pathway activation	[16,29]
	C3, C4 (complement)	Reduced levels indicate immune complex activation and may support flare prediction	Not assessed for molecular refractoriness	Do not reliably distinguish active inflammation from accrued damage	[29,30]
Pathway-associated biomarkers	IFN-α/IFN-β	Reflect activation of the interferon pathway and association with more severe phenotypes	Not validated for molecular refractoriness	Not validated as markers of persistent interferon pathway activation under therapy	[22,23]
	BAFF	Elevated in active SLE and linked to B-cell survival	Partial evidence; may predict response to B-cell-targeted therapy	Baseline elevation does not demonstrate BAFF-axis refractoriness	[24,25]
	IL-17/IL-23	Associated with lupus nephritis activity and non-response	Emerging evidence links persistent IL-23 activity to non-response in small cohorts	Not validated longitudinally under therapy	[26]
	Circulating NETs	Associated with endothelial dysfunction and vascular damage	Not assessed for molecular refractoriness	Downstream effector of inflammation rather than a marker of persistent pathway activation	[27,33]
Organ-resolved biomarkers	Anti-C1q	Associated with renal flares	Not assessed for molecular refractoriness	Organ-associated marker with limited mechanistic specificity	[34,35,36]
	Urinary MCP-1 (CCL2)	Reflects intrarenal inflammatory activity and may support disease monitoring	Associated with response monitoring in LN; not validated for molecular refractoriness	Cannot reliably distinguish active renal inflammation from chronic scarring	[31]
	Urinary soluble CD163	Reflects intrarenal macrophage activation; elevated in proliferative LN	Not assessed for molecular refractoriness	Correlates with severity rather than therapeutic failure	[37]
	Urinary TWEAK	Correlates with lupus nephritis activity and treatment response	Partial evidence; associated with response monitoring in LN, but not validated for molecular refractoriness	Activity/response marker not validated as a resistance marker	[38,39]
Candidate biomarkers of treatment response	IgA2 anti-dsDNA	Associated with response to sequential B-cell-targeted therapy; predicts belimumab response after rituximab	Yes; therapy-specific response stratification	Exploratory and not generalizable across treatment classes	[28]
	Interferon Gene Signature (IGS)	Associated with differential response to IFN receptor blockade (anifrolumab) and, in exploratory analyses, B-cell-targeted therapy	Partial evidence; therapy-specific stratification	Not validated as a marker of persistent interferon pathway activation under treatment	[30,40]

**Abbreviations:** SLE, systemic lupus erythematosus; D2T-SLE, difficult-to-treat systemic lupus erythematosus; anti-dsDNA, anti-double-stranded DNA antibodies; C3, complement component 3; C4, complement component 4; IFN-α, interferon-alpha; IFN-β, interferon-beta; BAFF, B-cell activating factor; IL, interleukin; NETs, neutrophil extracellular traps; MCP-1, monocyte chemoattractant protein-1; CCL2, C-C motif chemokine ligand 2; CD163, cluster of differentiation 163; TWEAK, tumor necrosis factor-like weak inducer of apoptosis; IgA2, immunoglobulin A2; LN, lupus nephritis; IGS, interferon gene signature.

**Table 2:** Proposed Immune Endotype Patterns in Difficult-to-Treat SLE.

Endotype Pattern	Dominant Circuit	Persistent Signal	Clinical Implication	Precision Therapy
Interferon-enriched/multi-interferon-associated	Interferon-responsive myeloid and lymphoid programs (type I with context-dependent type II/III contribution)	Interferon-stimulated gene modules (IGS), ideally interpreted alongside direct IFN measurements	Systemic flares	Candidate enrichment for IFN pathway inhibition (e.g., IFN-I blockade), not a definitive mechanistic assignment
B cell-driven	Plasmablast/autoantibody axis	Plasmablast module	Serological persistence; early relapse	B-cell/plasma cell-targeted therapy
Innate/NET-driven	Neutrophil/NET axis	NET gene signature	Vascular inflammation	Innate-pathway modulation
Cytotoxic T-cell-driven	CD8^+^ effector axis	Cytotoxic T-cell signature	Severe systemic phenotype	T-cell-directed strategies
Cytokine-/Th17-associated	IL-23/IL-17 inflammatory axis	IL-17/IL-23-associated transcriptional or cytokine signal	Renal/systemic inflammatory persistence	Cytokine-directed strategies (investigational)

**Abbreviations:** IFN, interferon; IFN-I, type I interferon; IGS, interferon gene signature; NET, neutrophil extracellular trap; CD8, cluster of differentiation 8; IL, interleukin; Th17, T helper 17. Note: Fibrosis- or remodeling-associated tissue signals are not included here as immune endotypes of molecular refractoriness, as they may reflect damage-associated or non-inflammatory states rather than persistent targetable immune activity.

## Data Availability

No new data were created or analyzed in this study. Data sharing is not applicable to this article.

## Supplementary Materials

The following supporting information can be downloaded at: https://www.mdpi.com/article/10.3390/ijms27094026/s1. References [58,59,60,61,62,63,64,65,66,67,68,69,70,71,72,73] are cited in the Supplementary Materials.

## Abbreviations

The following abbreviations are used in this manuscript:

ACR	American College of Rheumatology
AUC	Area under the curve
BAFF	B-cell activating factor
CAR-T	Chimeric antigen receptor T-cell therapy
CD8	Cluster of differentiation 8
CD19	Cluster of differentiation 19
CCL2	C-C motif chemokine ligand 2
BICLA	British Isles Lupus Assessment Group-based Composite Lupus Assessment
D2T-SLE	Difficult-To-Treat Systemic Lupus Erythematosus
DORIS	Definitions Of Remission In Systemic Lupus Erythematosus
EULAR	European Alliance of Associations for Rheumatology
GCS	Glucocorticosteroids
HCQ	Hydroxychloroquine
IFN	Interferon
IFN-I	Type I interferon
IGS	Interferon Gene Signature
IL	Interleukin
LLDAS	Lupus Low Disease Activity State
LN	Lupus nephritis
MCP-1	Monocyte chemoattractant protein-1
NET	Neutrophil extracellular traps
PD-1	Programmed cell death protein 1
PGA	Physician Global Assessment
SLE	Systemic lupus erythematosus
SLEDAI	Systemic Lupus Erythematosus Disease Activity Index
SRI-4	Systemic Lupus Erythematosus Responder Index 4
T2T	Treat-to-target
TWEAK	Tumor necrosis factor-like weak inducer of apoptosis
