# Supplementary material for: Redefining Difficult-to-Treat Systemic Lupus Erythematosus: Biomarkers of Molecular Refractoriness Beyond Clinical Failure
Source: Int J Mol Sci. 2026 Apr 30;27(9):4026. doi: 10.3390/ijms27094026 (PMC13163840; doi:10.3390/ijms27094026)
Supplement: Supplementary file 1 [file ijms-27-04026-s001.zip › ijms-4240993-supplementary.pdf]

International Journal of *Molecular Sciences*

MDPI

Supplementary Table S1. Expanded overview of currently available biomarkers in systemic lupus erythematosus and their limitations in the assessment of molecular refractoriness. The table provides a structured overview of systemic, pathway-associated, organ-specific, and treatment-response biomarkers in SLE, highlighting their principal biological and clinical significance, the extent to which they inform refractoriness assessment, and the major limitations preventing their current use as validated markers of persistent pathway activity in difficult-to-treat disease.

Domain	Biomarker	Principal Clinical/Biological Readout	Evidence for Refractoriness Assessment	Limitation in the D2T-SLE Context	References
	Anti-dsDNA	Reflects immune complex-driven systemic activity and correlates with overall disease burden, particularly in lupus nephritis	Not assessed for molecular refractoriness	Captures humoral autoimmune activity but does not define persistent pathogenic pathway activation under therapy	[16,29]
	C3, C4 (complement)	Reduced levels indicate complement consumption secondary to immune complex activation and may support flare prediction	Not assessed for molecular refractoriness	Reflect downstream immune complex activity but do not distinguish active inflammation from irreversible tissue damage or persistent immune dysregulation	[29,30]
**Systemic inflammatory activity**	IFN-α / IFN-β	Reflect activation of the type I interferon axis and are associated with immunologically active and clinically severe disease phenotypes	Not validated for molecular refractoriness	Not validated as longitudinal markers of persistent interferon pathway activation during ongoing targeted therapy	[22,23]
	BAFF	Reflects enhanced B-cell survival signaling and is elevated in active SLE	Partial evidence for therapy-specific stratification; may predict response to B-cell-targeted therapy	Baseline elevation does not establish persistent BAFF-dependent refractoriness or sustained B-cell axis dominance under treatment	[24,25]
	IL-6, IL-10, TNF-α	Reflect a proinflammatory cytokine milieu associated with high disease activity; IL-10 may also carry prognostic information	Not assessed for molecular refractoriness	Primarily indicate inflammatory burden rather than stable, pathway-specific molecular persistence	[58,59]
	IL-17 / IL-23	Reflect activation of the Th17-associated	Emerging evidence for	Lack longitudinal validation as	[26]

Domain	Biomarker	Principal Clinical/Biological Readout	Evidence for Refractoriness Assessment	Limitation in the D2T-SLE Context	References
		inflammatory axis and have been associated with lupus nephritis activity and treatment response in exploratory studies	association with non-response in small cohorts	markers of persistent IL-23/Th17-driven disease under therapeutic pressure	
	CXCL9 / CXCL10 / CXCL11	Reflect interferon-inducible chemokine activity associated with systemic flares and lupus nephritis	Not assessed for molecular refractoriness	Capture immune activation state but not biological resistance or persistent pathway dominance	[60]
	S100A8	Reflects innate immune activation and correlates with high disease activity and lupus nephritis	Not assessed for molecular refractoriness	Indicates inflammatory amplification rather than persistent, targetable pathway activity	[61]
	Epigenetic markers (miRNA, lncRNA)	Reflect transcriptional and post-transcriptional immune dysregulation associated with disease activity and organ involvement; potential diagnostic and prognostic biomarkers	Experimental	Lack standardization, reproducibility, and longitudinal validation for defining treatment-persistent molecular states	[62,63]
**Organ-specific biomarkers: lupus nephritis / renal activity**	Anti-C1q	Reflects renal-associated humoral immune activation and is associated with lupus nephritis flares	Not assessed for molecular refractoriness	Organ-enriched serological marker with limited mechanistic specificity for persistent therapeutic non-response	[34–36]
	Urinary MCP-1 (CCL2)	Reflects intrarenal chemokine-driven inflammation and may support monitoring of active lupus nephritis	Associated with response monitoring in LN; not validated for molecular refractoriness	Does not reliably distinguish active immune-mediated renal inflammation from chronic scarring or residual tissue injury	[31]
	Urinary NGAL	Reflects early renal injury and tubular stress	Not assessed for molecular refractoriness	Functions primarily as an injury marker rather than a marker of persistent pathogenic pathway activation	[64,65]

	Urinary soluble CD163	Reflects intrarenal macrophage activation and is elevated in proliferative lupus nephritis	Not assessed for molecular refractoriness	Correlates with inflammatory severity but has not been validated as an indicator of treatment-resistant renal immune activation	[37]
	Urinary adiponectin	Associated with active lupus nephritis and renal inflammatory involvement	Not assessed for molecular refractoriness	Limited mechanistic specificity and no validation as a marker of persistent renal pathway activity	[31]
	Urinary TWEAK	Reflects intrarenal inflammatory activity and correlates with lupus nephritis activity and treatment response	Partial evidence; associated with response monitoring in LN, but not validated for molecular refractoriness	Supports activity and response assessment but does not establish persistent resistance-associated molecular signaling	[38,39]
	VCAM-1 / ICAM-1	Reflect endothelial and inflammatory activation associated with renal histopathologic severity	Not assessed for molecular refractoriness	Adhesion-related inflammatory markers that do not define persistent pathway activation or therapeutic resistance	[66]
	ANCAs	Associated with worse renal outcomes in selected patients	Not assessed for molecular refractoriness	Primarily prognostic rather than mechanistically informative for refractory immune persistence	[67]
**Organ-specific biomarkers: neuropsychiatric SLE**	Anti-ribosomal P	Associated particularly with psychiatric manifestations of neuropsychiatric SLE	Not assessed for molecular refractoriness	Limited specificity and no evidence for defining persistent CNS-directed immune activation under therapy	[68]
	Anti-Sm, anti-Ro, antiphospholipid antibodies	Associated with selected CNS manifestations, particularly thrombotic events in aPL-positive patients	Not assessed for molecular refractoriness	Associations are heterogeneous and do not establish a coherent mechanism of persistent neuroimmune refractoriness	[69]

	Anti-NMDAR (CSF)	Reflects CNS-associated autoantibody activity and is associated with neuropsychiatric involvement	Not assessed for molecular refractoriness	Does not reliably distinguish inflammatory neuropsychiatric disease from vascular or secondary mechanisms	[70,71]
	TCN2, KLK5, CST6, Trappin-2, and L-selectin in CSF	Candidate CSF biomarkers reflecting compartmentalized CNS immune activity associated with NPSLE	Experimental	Lack validation in longitudinal therapeutic studies and no evidence for resistance-specific utility	[72]
**Organ-specific biomarkers: cardiovascular immune injury**	LDGs	Reflect neutrophil dysregulation, enhanced NET formation, and endothelial injury	Not assessed for molecular refractoriness	Capture vascular immune injury rather than treatment-persistent lupus-specific molecular refractoriness	[33]
	Circulating NETs	Reflect neutrophil effector activation associated with endothelial dysfunction and vascular damage	Not assessed for molecular refractoriness	Represent a downstream inflammatory effector state rather than a validated marker of persistent pathway-specific resistance	[27,33]
	VAP-1	Associated with vascular inflammation and atherosclerotic burden	Not assessed for molecular refractoriness	Cardiovascular complication marker without established relevance to treatment-resistant immune pathway persistence	[73]
	PTX3	Reflects vascular inflammatory burden and correlates with inflammation and glucocorticoid exposure	Not assessed for molecular refractoriness	Indicates vascular inflammation rather than disease-specific molecular refractoriness	[73]
	TRAIL-R2	Associated with atherosclerotic involvement and vascular injury	Not assessed for molecular refractoriness	Complication-associated marker without demonstrated utility in defining persistent immune pathway activation	[73]

**Candidate biomarkers of treatment response**	IgA2 anti-dsDNA	Associated with response to sequential B-cell-targeted therapy and predicts belimumab response after rituximab	Yes; therapy-specific response stratification	Exploratory biomarker not yet generalizable across therapeutic classes or validated as a marker of stable molecular refractoriness	[28]
	Interferon Gene Signature (IGS)	Reflects transcriptional activation of the interferon pathway and is associated with differential response to IFN receptor blockade and, in exploratory analyses, selected B-cell-targeted therapies	Partial evidence; therapy-specific stratification	Not validated as a longitudinal biomarker of persistent interferon pathway activity under treatment	[30,40]

Abbreviations: SLE, systemic lupus erythematosus; D2T-SLE, difficult-to-treat systemic lupus erythematosus; anti-dsDNA, anti-double-stranded DNA antibodies; C3, complement component 3; C4, complement component 4; IFN-α, interferon-alpha; IFN-β, interferon-beta; BAFF, B-cell activating factor; IL, interleukin; TNF-α, tumor necrosis factor-alpha; Th17, T helper 17; CXCL, C-X-C motif chemokine ligand; miRNA, microRNA; lncRNA, long non-coding RNA; MCP-1, monocyte chemoattractant protein-1; CCL2, C-C motif chemokine ligand 2; LN, lupus nephritis; NGAL, neutrophil gelatinase-associated lipocalin; CD163, cluster of differentiation 163; TWEAK, tumor necrosis factor-like weak inducer of apoptosis; VCAM-1, vascular cell adhesion molecule 1; ICAM-1, intercellular adhesion molecule 1; ANCA, anti-neutrophil cytoplasmic antibody; CNS, central nervous system; aPL, antiphospholipid antibodies; NMDAR, N-methyl-D-aspartate receptor; CSF, cerebrospinal fluid; NPSLE, neuropsychiatric systemic lupus erythematosus; TCN2, transcobalamin 2; KLK5, kallikrein-related peptidase 5; CST6, cystatin E/M; LDGs, low-density granulocytes; NETs, neutrophil extracellular traps; VAP-1, vascular adhesion protein-1; PTX3, pentraxin 3; TRAIL-R2, TNF-related apoptosis-inducing ligand receptor 2; IgA2, immunoglobulin A2; IGS, interferon gene signature.
